# Systems biological approach on neurological disorders: a novel molecular connectivity to aging and psychiatric diseases

**DOI:** 10.1186/1752-0509-5-6

**Published:** 2011-01-12

**Authors:** Shiek SSJ Ahmed, Abdul R Ahameethunisa, Winkins Santosh, Srinivasa Chakravarthy, Suresh Kumar

**Affiliations:** 1 Department of Biotechnology, School of Bioengineering, SRM University, Kattankulathur -603 203, Tamil Nadu, India; 2 Computational Neuroscience Laboratory, Department of Biotechnology, Indian Institute of Technology Madras, Chennai -600 036, Tamil Nadu, India; 3 Department of Bioinformatics, School of Bioengineering, SRM University, Kattankulathur -603 203, Tamil Nadu, India; 4 Department of Neurology, SRM Medical College Hospital and Research Centre, Kattankulathur -603 203, Tamil Nadu, India

## Abstract

**Background:**

Systems biological approach of molecular connectivity map has reached to a great interest to understand the gene functional similarities between the diseases. In this study, we developed a computational framework to build molecular connectivity maps by integrating mutated and differentially expressed genes of neurological and psychiatric diseases to determine its relationship with aging.

**Results:**

The systematic large-scale analyses of 124 human diseases create three classes of molecular connectivity maps. *First*, molecular interaction of disease protein network generates 3632 proteins with 6172 interactions, which determines the common genes/proteins between diseases. *Second*, Disease-disease network includes 4845 positively scored disease-disease relationships. The comparison of these disease-disease pairs with Medical Subject Headings (MeSH) classification tree suggests 25% of the disease-disease pairs were in same disease area. The remaining can be a novel disease-disease relationship based on gene/protein similarity. Inclusion of aging genes set showed 79 neurological and 20 psychiatric diseases have the strong association with aging. *Third and lastly*, a curated disease biomarker network was created by relating the proteins/genes in specific disease contexts, such analysis showed 73 markers for 24 diseases. Further, the overall quality of the results was achieved by a series of statistical methods, to avoid insignificant data in biological networks.

**Conclusions:**

This study improves the understanding of the complex interactions that occur between neurological and psychiatric diseases with aging, which lead to determine the diagnostic markers. Also, the disease-disease association results could be helpful to determine the symptom relationships between neurological and psychiatric diseases. Together, our study presents many research opportunities in post-genomic biomarkers development.

## Background

Systems biology is an indispensable approach to study the complex mechanisms of any disease or disorders. After post-genomic era the accumulation of genomics and proteomics data are widely flooded. However, there is an unrealized opportunity remains in the understanding of detailed molecular mechanisms of several neurological disorders [1,2]. Thus, the molecular diagnosis of most of the neurological disorders remains difficult and mostly carried out by neurological examination [3]. The current molecular connectivity approaches of systems biology are mainly focusing on building large protein networks without probing the interaction mechanisms specific to disorders or disease condition [4,5]. Hence, the possibility of finding successful biomarkers through systems biology approach is intricate. In order to gain a better understanding of molecular mechanism, disease relationship and biomarkers, the genes implicated within similar disorders are need to be focused.

The systems biological concepts of disease interaction were usually made by collecting signature genes of genetically heterogeneous hereditary diseases and investigating the different mutations in a same gene (allelic heterogeneity) giving rise to different disorders [6]. Similar, trends are followed for differentially regulating genes and linking them to various diseases [7]. Here, we had taken an integrated approach of mutated and differentially regulating genes and exploring diseasome network that corresponds to the neurological and psychiatric diseases. Such integrative approach will improve the confidence of finding specific markers for diseases. The reasons that we choose an integrative approach on neurological disorders are two-fold. *First*, the understanding of neurological disorder is considerably less, because of difficulty in obtaining brain tissue for many cases. *Second*, there is an increasing prevalence rate [8,9] and lack of molecular diagnosis for most of the neurological disorders [10,11].

In this study, we propose an integrative, network-based model of mutated and differentially regulating genes of 100 neurological and 24 psychiatric diseases (see Additional File 1 for a disease category), that identifies the neurological and psychiatric relationship and their association with aging. Furthermore, this network model helps to understand the common mechanism between diseases through common pathway network (CPN). Overall, our findings highlight the importance of integrating the gene/protein data of neurological diseases into future molecular biomarkers and drug target discovery.

## Results and Discussion

**Figure 1:**
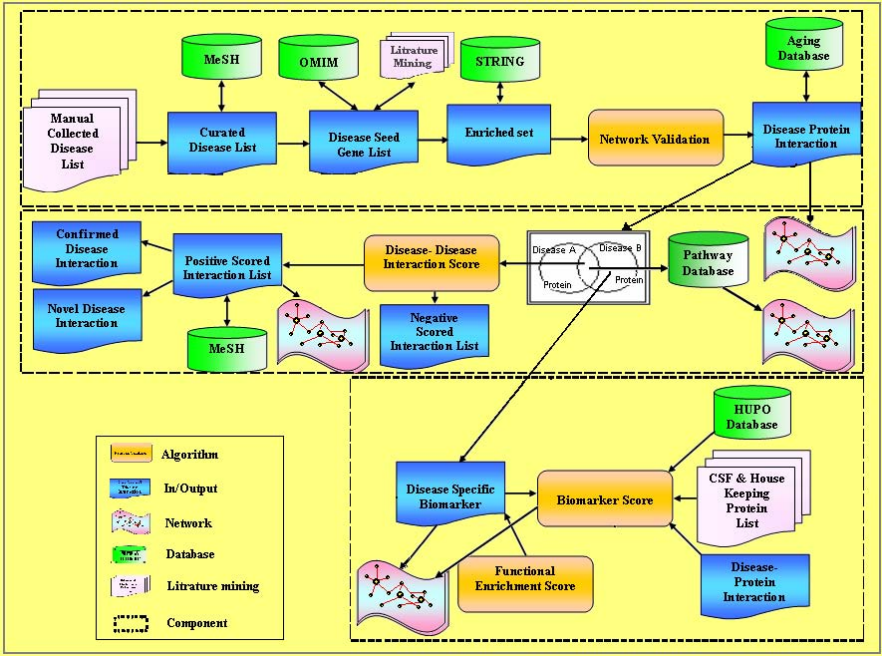
**Computational framework for developing molecular connectivity maps**. The framework consists of three major components: disease protein network, disease-disease network and disease biomarker network. The first component takes the inputs from database and literature and outputs a disease protein network (DPN). The second component takes the input from DPN and generates the output of positively scored disease-disease network (DDN) using scoring algorithm. Further, the second component was used to generate sub-component of common pathway network (CPN). The final disease biomarker network (DBN) component was generated from DPN showing proteins specific to diseases.

**Figure 2:**
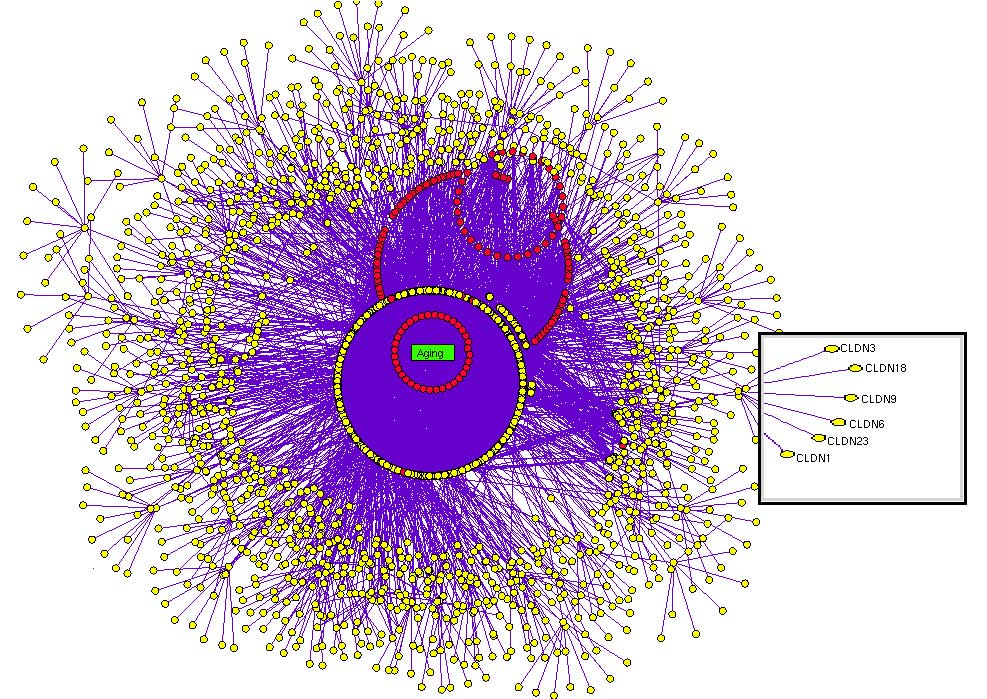
**Disease protein network (DPN)**. In DPN each nodes (seed and enriched proteins) were colored yellow and the aging genes were colored as red and the proteins interactions were represented in violet solid lines.

**Figure 3:**
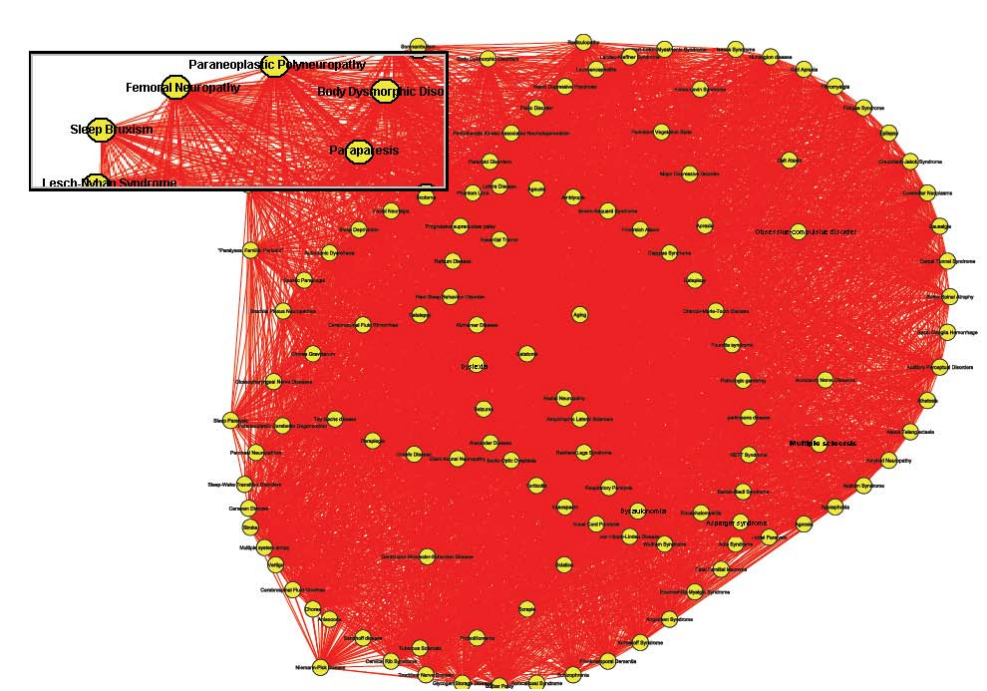
**Disease-disease network (DDN)**. In disease-disease network, each node represents to a disease yellow colored. Two diseases were connected by red solid line, if they attained the positive score in algorithm. The total of 4845 positively scored disease-disease interactions were shown along with the aging interactions.

**Figure 4:**
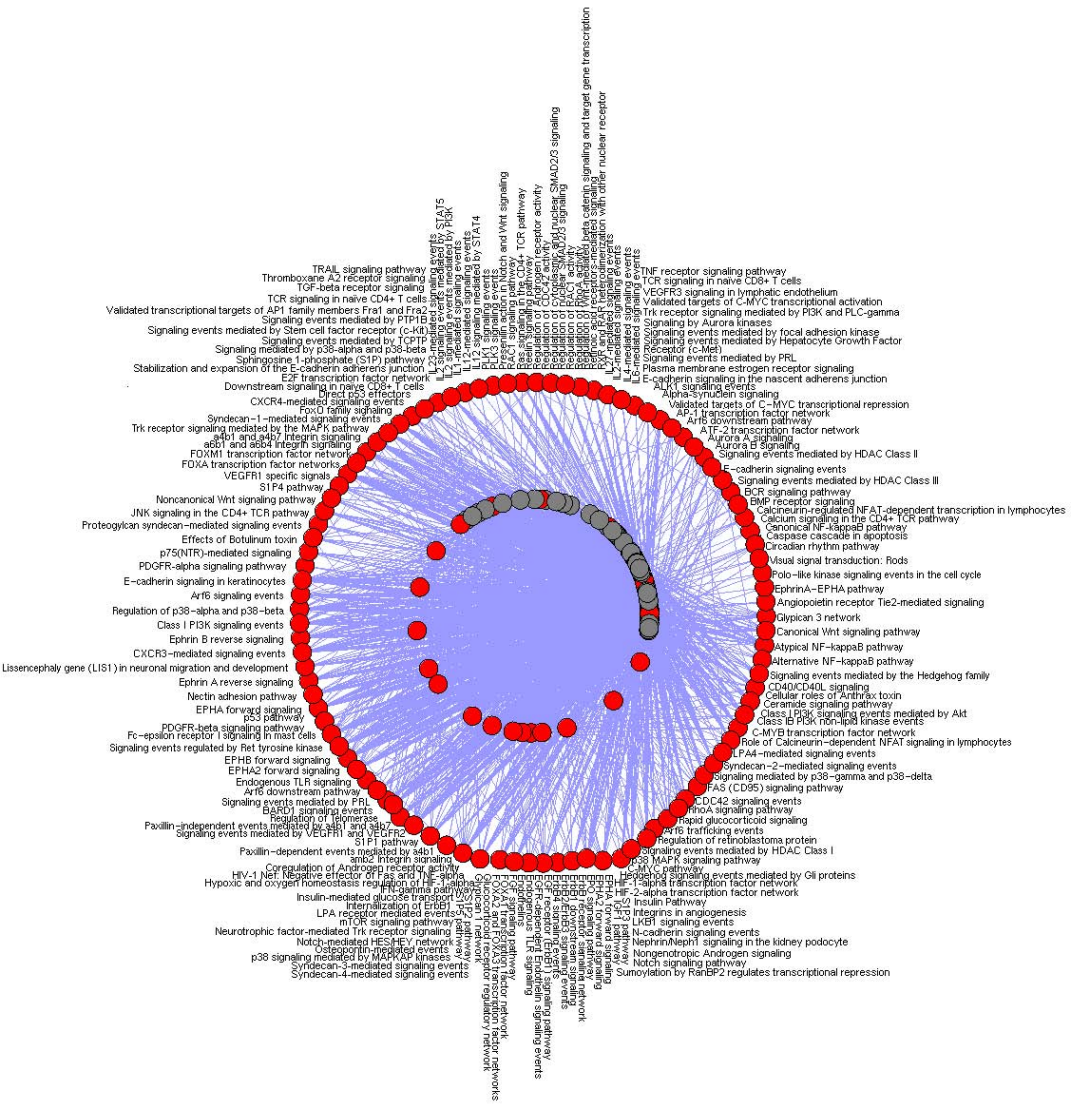
**Common pathway network (CPN)**. In CPN, node represents to a disease (gray) and their associated pathway represented in red. Two diseases were connected to a pathway, if both the disease shares proteins/genes that are associated to a pathway.

In this study, we developed a novel computational framework (Figure. 1) to build disease-protein network (DPN) (Figure. 2), disease-disease network (DDN) (Figure. 3) and common pathway molecular network (CPN) (Figure. 4). Our approach of integrating mutated and differentially expressed diseases genes allow us to validate the neurological and psychiatric relationships with aging. In addition, this approach helps to predict the disease specific biomarkers for the potential diagnosis. We showed that this approach was effective in constructing a statistically significant molecular connectivity map of 124 diseases with 3632 proteins. This work pointed out a new direction for biomedical researchers to investigate the molecular interaction network with the known dysfunctional genes to identify disease relationship. The results of disease-disease connectivity map constructed from disease protein interactions helps to guide the hypothesis for generation of biomarkers for neurological and psychiatric diseases.

**Figure 5:**
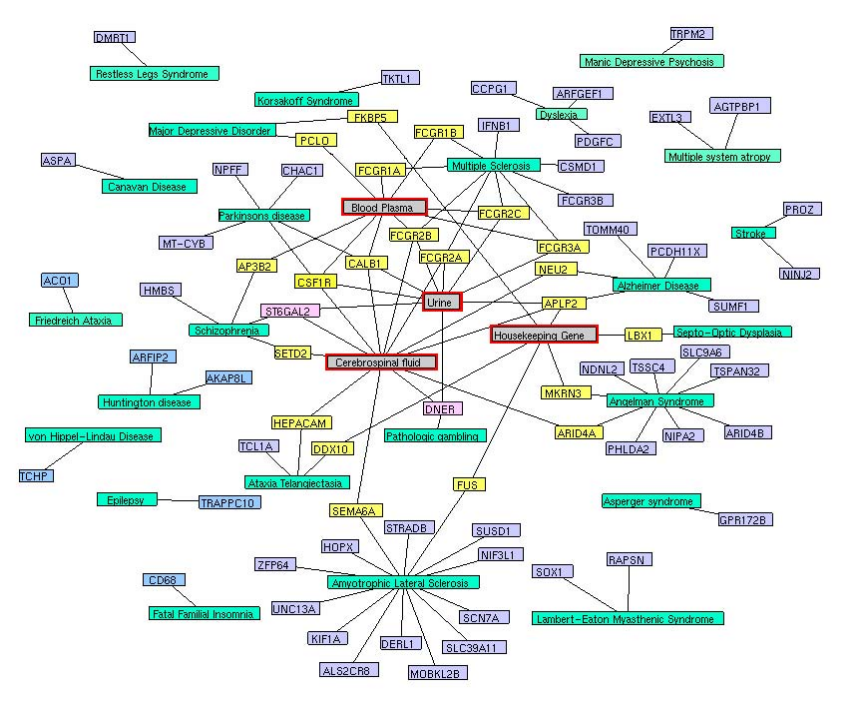
**Disease biomarker network (DBN)**. The disease biomarker network contains 24 diseases (green) with 73 biomarkers. The biomarkers were colored based on the diagnostic parameters (gray). The associations of biomarkers with any of the diagnosis parameters (gray) are represented in yellow, while other biomarkers are indicated in violet.

**Figure 6:**
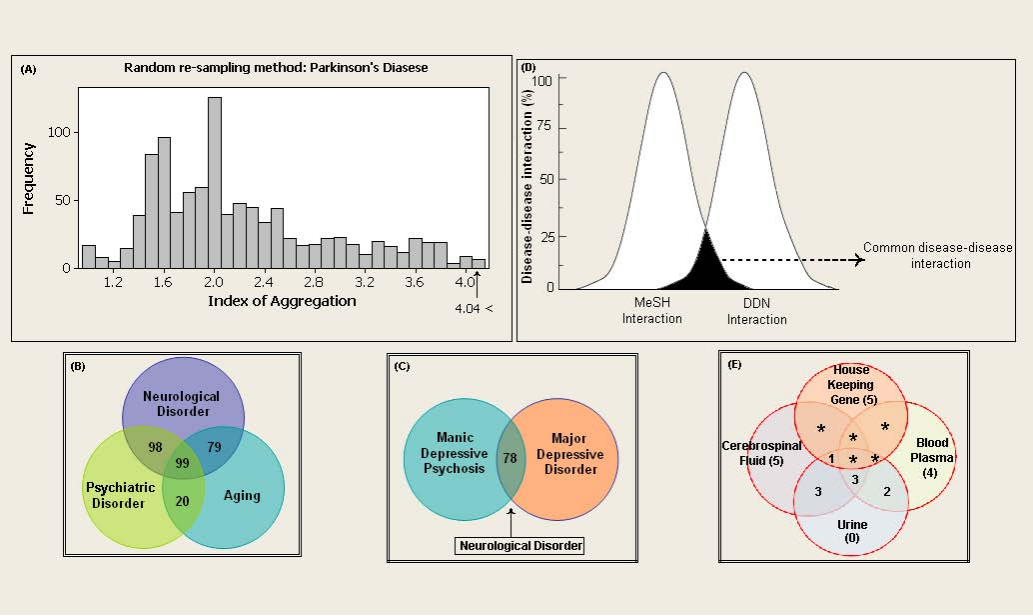
**Characterizing the disease modules**. (**a**) Histogram of the index of aggregation distribution for Parkinson's disease enriched sets of proteins randomly selected from a database. The arrow indicates the aggregation values for the enriched Parkinson's disease proteins set. The Venn diagram (**b**) showed the neurological diseases relationships between aging and psychiatric diseases, The Venn diagram (**c**) showing the neurological disease relationship with depression. (**d**) Peak representation of positively scored disease pairs category and MeSH disease pairs category. The common region indicates the similarity disease pairs between the two categories. The Venn diagram (**e**) shows the presences of biomarkers in biofluid and house keeping genes.

We used OMIM and literature mining to generate the initial list of 1211 seed genes for 124 diseases. Using STRING, we expanded 1211 seed genes/proteins to 13011 human proteins with 11800 proteins as enriched set. Of 13011 proteins, most of the proteins were associated to one or more diseases showing the possibility of successful interactions between the diseases. These records were further mapped to HGNC database to obtain a unique gene symbol, to avoid false interactions. As explained in the methodology, the disease protein network (DPN) was constructed to have 3632 proteins with 6172 interactions (see Additional File 2 for protein interaction). In addition, we included the 261 ageing genes to the DPN, to make a valid correlation of aging within the analyzed diseases. These aging genes were presumably more interesting to determine the association of aging with neurological and psychiatric diseases. This final DPN containing 3999 proteins with 6557 interaction (Figure. 2) was important to generate the disease-disease relationship (Figure. 3), common disease pathway network (Figure. 4) and disease biomarker network (Figure. 5). In Figure 2, we showed the curated view of seed and enriched set of proteins interactions including aging genes/proteins. All proteins were shown as nodes; the seed and enriched proteins are colored yellow and the aging genes were colored as red. Similarly, in Figure 3, nodes indicate disease and edges indicate the link between diseases. The disease-disease interaction was comprehended but the reliability of the DDN depends on DPN. Therefore, the overall proteins involved in the DPN were validated by analyzing its significance by a random sampling method. For instance, the protein sub-network (PSN) of Parkinson's disease contains 297 proteins, in which *PSEN1 *is highly connected protein, showed 12 interactions in its network. Therefore, the index of aggregation was calculated as 4.04. The random sampling method was carried out as described in the methodology. Only seven runs out of 1000 resulted in an index of aggregation value greater than 4.04 (Figure. 6A). Therefore, the *p*-*value *of the observed index of aggregation of the Parkinson's disease network was 0.007. Similar trends were followed for all the diseases and geometric mean for overall *p-values *was calculated as 0.00612. With the significance of disease-protein interaction data, the DDN was generated in order to determine the relationship between the diseases. Two diseases were connected by a link if same proteins/genes were implicated in both the diseases. These identified disease-disease interactions were further validated by interaction score. This process generated a total of 4845 positively scored disease-disease interactions (Additional File 3 for positively scored interaction). In these identified interactions, 79 neurological and 20 psychiatric diseases were shown to have a strong association with aging (Figure. 6B) (see Additional File 4 for aging interaction). Further, the analyses of 100 neurological diseases revels 98 diseases were shown to have relationships with any of the analyzed psychiatric diseases. For example, 78 neurological diseases provide the common association with both major depressive disorder and manic depressive psychosis, suggesting the role of depressive state in these 78 diseases (Figure. 6C). To access the reliability of these connections, we mapped the connected disease pair onto MeSH term. Of 4746 positively scored disease-disease links excluding aging interactions, 1219 (25%) pair shared common disease term (see Additional File 5 for MeSH validated interaction), (Figure. 6D). For example, Alzheimer's and Parkinson's disease were present in the neurodegenerative disease section of the MeSH tree. The remaining 3527 disease pairs were not located in the same branch of MeSH tree. However, these positively scored disease connections that located in different branches of MeSH tree was particularly interesting, because they provide novel disease relationships that were primarily relying on gene similarity instead of phenotypic classification. For example, Parkinson's disease has been connected to REM sleep behavior disorder, not surprisingly, many studies indicate the association of REM sleep behavior disorder with Parkinson's disease [12-14]. However, they were not explicitly in same disease branch according to MeSH. For better understanding of common mechanism between the diseases, the proteins/genes that commonly associated between each disease pairs were mapped to NCI-Nature Pathway Interaction Database [15]. This process generates 179 associated pathways between the disease pairs (Additional File 6 for common pathway network). Further, analyses of these pathways may guide for the drug target discovery. For instance, our study showed the association of glucocorticoid receptor regulatory network between Alzheimer's and major depressive disorder. Supportive to this result, previous study of Filippo *et al*., suggests glucocorticoid receptor can be the common drug target for both Alzheimer's and major depressive disorder [16].

Biomarkers are the most interesting part of any biomedical research, and it is essential for neurological and psychiatric diseases because most of these diseases lack diagnostic markers. Every disease was expected to have its own fingerprint, which subsequently helps in detection of diseases. Though, we analyzed 124 diseases, only 24 diseases were shown to have a disease specific biomarkers (Figure. 5) (Additional File 7 for biomarkers list) while, others may have shared their fingerprint with their related diseases. Interestingly, few of our identified biomarkers were previously reported. For instance, our previous study suggests that pyruvate dehydrogenase lipoamide beta (*PDHB*) and neuropeptide FF-amide peptide precursor (*NPFF*) are the biomarkers for Parkinson's disease [17]. However, this approach provides the additional information that *PDHB *is not only associated with Parkinson's disease but also associated with Athetosis and Friedreich Ataxia, whereas *NPFF *was found unique to Parkinson's disease, suggesting the possibility as biomarker. The significance of these disease specific biomarkers was validated by enrichment score based on gene ontology with a threshold of 1.3. All the identified disease biomarkers passed the threshold and confirmed its significance to its diseases. Furthermore, the identified biomarkers of each disease was scored based on the feasibility of diagnosis from biofluids, this analysis would be of marginal interest to researchers focusing on diagnosis of these 24 diseases from biofluids. Each parameter such as house keeping genes and biofluids circulating proteins were assigned a value (m-score) to generate the overall diagnostic score. In comparison with other biofluids, urine has two characteristics feature that makes it a preferred high m-score value of 0.7 for feasible diagnosis. *First*, urine can be obtained in large quantities using non-invasive procedures. This allows repeated sampling of the same individual for disease surveillance. *Second*, the urinary protein content is relatively stable probably due to the fact that urine "stagnates" for hours in the bladder [18]. However, the reliability of diagnostic biomarkers in CSF is high because, it has direct contacts with the extracellular space of the brain, making it as a unique medium in detecting biochemical changes in the central nervous system. However, obtaining the CSF samples is difficult thereby it was assigned to a least diagnostic m-score of 0.3. Considering the feasibility of both urine and CSF, the average m-score of 0.5 was assigned to biomarkers presence in blood plasma. Of 73 identified biomarkers proteins, 18 were found to be present in any one of the biofluids and three biomarkers were identified to be circulating in all the biofluids (Figure. 6E). Further comparison of biomarkers with house keeping genes, showed six biomarkers proteins were encoded by essential genes, which enhances the possibility of diagnosis in any tissue. Though, we suggest these top scored proteins as feasible diagnostic markers (Figure. 5) (Table. 1), further studies are need to be carried out to determine its significance as biomarkers.

**Table 1:** Biomarkers score

Disease	Biomarkers (score)
Alzheimer's Disease	*APLP2 *(4), *NEU2 *(1.4), *PCDH11X *(0.5), *SUMF1 *(0.5), *TOMM40 *(0.5)

Amyotrophic Lateral Sclerosis	*ALS2CR8 *(0.5), *DERL1 *(0.5), *FUS *(1), *HOPX *(0.5), *KIF1A *(0.5), ***MOBKL2B ***(0.5), *NIF3L1 *(0.5), *SCN7A *(0.5), *SEMA6A *(1.4), ***SLC39A11 ***(0.5), *STRADB *(0.5), ***SUSD1 ***(0.5), ***UNC13A ***(0.5), ***ZFP64 ***(0.5)

Angelman Syndrome	*ARID4A *(1.4), *ARID4B *(0.5), *MKRN3 *(1), *NDNL2 *(0.5), *NIPA2 *(0.5), *PHLDA2 *(0.5), ***SLC9A6 ***(0.5), *TSPAN32 *(0.5), *TSSC4 *(0.5)

Asperger Syndrome	*GPR172B *(0.5)

Ataxia Telangiectasia	*DDX10 *(1), *HEPACAM *(1.4), *TCL1A *(0.5)

Canavan Disease	***ASPA ***(0.5)

Dyslexia	*ARFGEF1 *(0.5), *CCPG1 *(0.5), *PDGFC *(0.5)

Epilepsy	*TRAPPC10 *(0.5)

Fatal Familial Insomnia	*CD68 *(0.5)

Friedreich Ataxia	*ACO1 *(0.5)

Huntington disease	*AKAP8L *(0.5), *ARFIP2 *(0.5)

Korsakoff Syndrome	*TKTL1 *(0.5)

Lambert-Eaton Myasthenic Syndrome	*RAPSN *(0.5), *SOX1 *(0.5)

Major Depressive Disorder	*FKBP5 *(1), *PCLO *(2)

Manic Depressive Psychosis	***TRPM2 ***(0.5)

Multiple Sclerosis	*CSMD1 *(0.5), *FCGR1A *(2), *FCGR1B *(2), *FCGR2A *(3.5), *FCGR2B *(5), *FCGR2C *(4.1), *FCGR3A *(4.1), *FCGR3B *(0.5), *IFNB1 *(0.5)

Multiple System Atropy	*AGTPBP1 *(0.5), *EXTL3 *(0.5)

Parkinson's Disease	***CALB1***(5), *CSF1R *(5), ***MT-CYB ***(0.5), *CHAC1 *(0.5), *NPFF *(0.5)

Pathologic gambling	*DNER *(3.5)

Restless Legs Syndrome	*DMRT1 *(0.5)

Schizophrenia	***AP3B2 ***(2), *HMBS *(0.5), ***SETD2 ***(1.4), ***ST6GAL2 ***(3.5)

Septo-Optic Dysplasia	*LBX1 *(1)

Stroke	***NINJ2 ***(0.5), *PROZ *(0.5)

*von Hippel-Lindau Disease*	*TCHP *(0.5)

The biomarkers of 24 diseases were indicated along with their diagnostic score. The biomarkers that are shown to be specific to neurological and psychiatric diseases in comparison with GWAS database are indicated in bold.

### Cross-validation of network

To validate our computational approach, the results obtained from this study were compared with the results of Goni *et al *and Goh *et al *approaches [19,4]. Our result was in agreement with Goni *et al *studies showing the successful interaction between Alzheimer's disease and multiple sclerosis. In addition to our result, several other studies also confirm the molecular relationship between Alzheimer's disease and multiple sclerosis [20-22]. However, similar interaction trend was not been achieved with Goh et al approach. This is because Goh et al approach of molecular connectivity was carried out on mutated genes, while our approach uses both differentially expressed and mutated disease genes for the generation of DDN. Hence, our approach confirms the effectiveness of integrating differential and mutated genes for reliable disease-disease relationships. On the other hand, the proposed biomarkers of our study were cross-validated using genetic association database (GWAS) [23] to confirm its disease specificity in context to neurological or psychiatric diseases. In our identified 73 biomarkers, only 27 biomarkers were shown to have disease association information, while the information of 46 biomarkers was not available in GWAS database. This is because the genetic associations of few diseases were not been included in GWAS database. However, the precision rate (PPV) was calculated only on these 27 biomarkers. All 27 biomarkers were confirmed to be specific to its diseases in context to the analyzed disorders. Hence, the PPV was calculated to be 100%.

### Limitations

Though, our present approach provides good accuracy in determining the disease-disease interaction and biomarkers, it has limitation in the aspects of biomarkers detection. In medicine, biomarkers are the molecules, specific to its pathological condition. Since, our study is focused on neurological and psychiatric diseases the obtained biomarkers are specific to its diseases of neurological and psychiatric disorders. However, there is a possibility for these 73 biomarkers to have an association with other disorders irrespective neurological and psychiatric diseases. Such limitation can be avoided by including all the disorders in a network and implementing our biomarker strategy for detection of biomarkers. However, with the available information of these 27 biomarkers, we validated across GWAS database. The results confirm that 15 biomarkers are specific to its disease and have no association with any other disorders (Table. 1).

## Conclusions

In conclusion, the disease-disease relationships are of great interest because such knowledge not only enhances our understanding of disease mechanisms, but also accelerates many aspects of biomarker and drug target discovery. These results can be interesting to neurologists, and our method can be generalized to other disease biology areas for systems biological investigation. We believe our approach to understand the mechanism involved in neurological disease has given a valuable insight into the relationship of aging and psychiatric illness. Moreover, these combined efforts resulted in identification of biomarkers that will greatly improve in diagnosis of neurological and psychiatric diseases.

## Methods

### Initial collection of disease related genes

**Figure 7:**
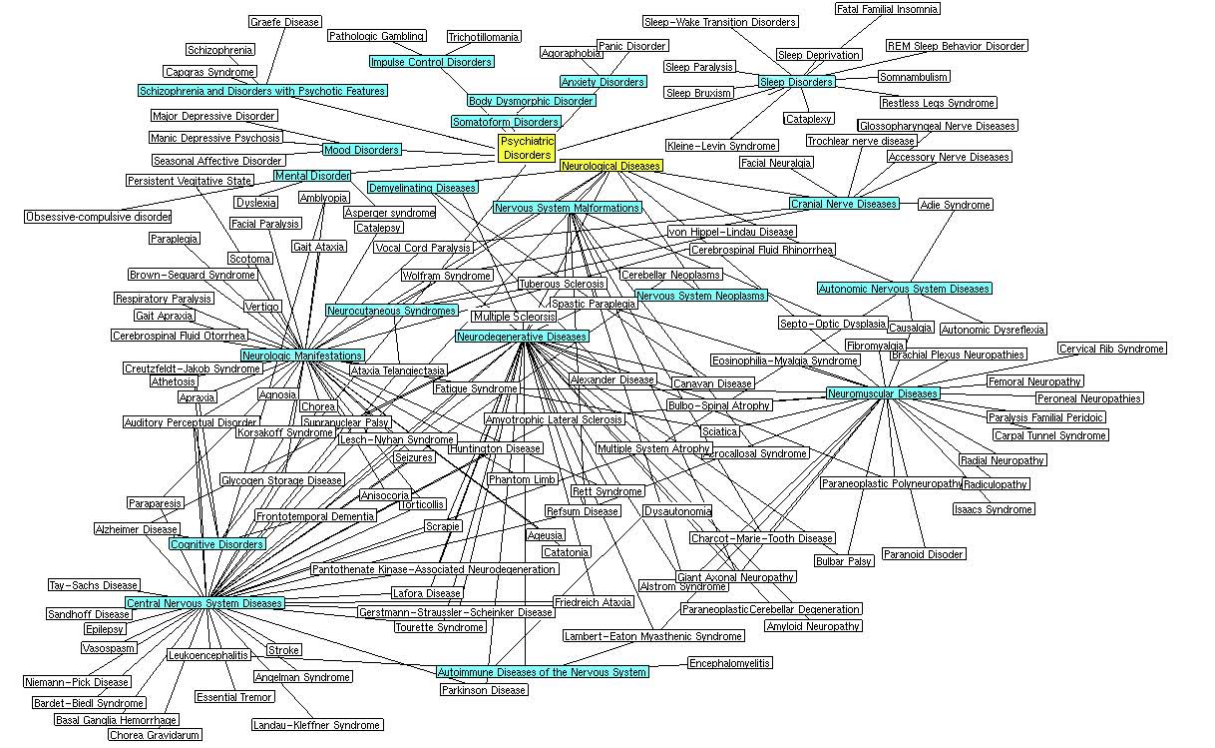
**MeSH based disease classification of 124 diseases**. The manually collected 124 diseases represented in white blocks were grouped based on the MeSH disease category (blue block) of neurological and psychiatric diseases (yellow block). Most of the diseases were linked to one or more MeSH disease categories. The overall linkage between the diseases was represented by solid lines.

The initial 124 disease list was manually collected and validated against the Medical Subject Headings (MeSH) database [24] in order to determine its neurological and psychiatric relationship. Of 124 diseases 100 have shown the relationship with neurological and 24 with psychiatric diseases (Figure. 7). These 124 diseases were taken as the basis for developing disease protein network. The network was constructed by retrieving the genes related to these diseases from Online Mendelian Inheritance in Man (OMIM) database [25] and literature mining. The human mutated genes were retrieved from OMIM and literature mining was carried out to retrieve the genes that are differentially expressed in its corresponding diseases. Overall, 1209 seed genes were retrieved and most of these genes were common to one or more diseases. Further, 261 human aging genes were included to this study to identify the association of aging to the analyzed diseases. This aging gene set was downloaded from GenAge database [26].

### Enriched protein network

The Search Tool for Retrieval of Interacting Genes/Protein (STRING) database [27] was used to collect protein interaction data to construct disease-protein network (DPN) from 1209 seed genes. The STRING database contains experimental and predicted protein interaction data of 630 organisms of both eukaryotes and prokaryotes. This study includes both experimental and predicted interaction of human proteins for the generation of disease-protein network, considering the successfulness of predicted interactions in several disease interaction studies [5,28]. To build disease-protein network, we pulled out proteins that are interacting to seed genes/proteins, with confidence scores ranging from 0.5 to 1.0. Such expanded set of initial seed proteins were denoted as enriched protein set and the interaction of seed and enriched set of each disease is known as protein sub-network (PSN). The aging genes set were included to the network without enrichment to make a strong correlation with neurological and psychiatric diseases. All genes were mapped to the official gene symbol using HUGO Gene Nomenclature Committee (HGNC) [29] to avoid false interaction to same genes/proteins and the data curation was carried out using Microsoft Excel and Microsoft Access. From these non-redundant interaction data, disease-protein network (DPN), disease-disease network (DDN), common pathway network (CPN) and disease-biomarker network (DBN) were created and visualized using Cytoscape version 2.7.0 and NAViGaTOR version 2.1software. In DPN, node represents disease proteins. The proteins of two diseases were connected if same proteins are associated with both diseases. In DDN, node represents disease, two diseases are connected to one another if they share at least one protein common to both the disease. Further, CPN was created from the commonly associated genes/protein between the disease pair and DBN was created by pulling out the disease specific seed proteins from DPN.

### Statistical significance of network

To validate the DPN, we adopted a similar method developed by Chen *et al *[28]. The index of aggregation was calculated for each PSN and their significance was evaluated by random re-sampling method. The largest connected protein in each PSN was selected and the *index of aggregation *for each PSN was calculated.

$$\text{Index of aggregation}(\%)=\frac{\begin{array}{c} \text{Largest connected}\\ \text{protein in protein sub-network} \end{array}}{\begin{array}{c} \text{Total number of}\\ \text{proteins in its protein sub-network} \end{array}}$$

In order to determine significance of DPN, the following random sampling method was executed,

1. Randomly select same number of seed proteins as in each PSN from Brain Gene Expression Map database [30].

2. Pull out the enriched set for the randomly selected seed proteins from STRING database.

3. Compute an *index of aggregation*.

4. Repeat the above steps for 1000 times to generate *index of aggregation*.

5. Compare the *index of aggregation *of protein-sub network with the distribution of previous steps, to calculate *p-value*.

6. Similarly, repeat the above steps for remaining PSN.

7. Finally, compute the geometric mean to the obtained *p-values *of 124 PSN.

### Disease-disease interaction score

The interaction score was assigned for each disease pair (Φ_dij_). The score indicates the strength of the interaction between the diseases based on the protein interaction.

$$\Phi_{\text{dij}}={\text{log (P}}_{\text{ij}}*N+\text{Z)}–{\text{log (P}}_{\text{i}}*{\text{P}}_{\text{j}}+Z\text{)}$$

Here, P_i _and P_j _are the total number of proteins for the disease, i and j, respectively. P_ij _is the total number of common protein between the two diseases. *N *is the size of entire proteins involved in the disease protein network. *Z *is a constant (*Z *= 1) introduced to avoid out-of bound errors, if P_i _= P_j _= P_ij _= 0. The expected result of Φ_dij _is positive, when the disease pair is over-represented and negative, when the disease pair is under-represented.

### MeSH based disease interaction mapping

Medical Subject Headings (MeSH) is the National Library of Medicine's controlled vocabulary thesaurus. It consists of sets of terms naming descriptors in a hierarchical structure that permits searching at various levels of specificity. We downloaded the disease tree file from MeSH, which contains 16 categories, including disease, chemicals and drug category, etc. The neurological disease category (C10) was classified into 15 major clusters and psychiatric disorder (F03) was classified into 16 major clusters. Each positively scored disease pair (Φ_dij_) was mapped to the neurological and psychiatric disease category to determine the reliability of disease connectivity. For instance, if each disease pair presents in single major cluster suggest having strong connectivity.

### Common Pathway network

In order to understand the common molecular mechanism between diseases, the proteins/genes that associated between each disease pair of disease-disease interaction were mapped to the NCI-Nature Pathway Interaction Database (PID) [15]. PID is a manually curated human pathway database contains 116 human pathways with 6180 interactions. PID provides the *p-value *based on the probability of occurrence of the proteins in the defined pathway. Lower the *p-value *the greater the probability of proteins associated towards a given pathway. Hence, we filtered the common pathway between the diseases by *p-value 0.05*.

### Biomarker's identification

The analysis of DPN was carried out to determine the biomarkers for each disease involved in this study. Biomarkers were identified by finding the disease specific seed proteins from the DPN network. This process was carried out by comparing the each seed protein of one PSN with the other PSN. If the seed protein was unique to its PSN, then the identified seed protein was considered as a biomarker (pi) to its disease.

### Significant enrichment biomarkers score

The functional enriched biomarkers score for each disease was computed based on the gene ontology. The scores were calculated using Biological Network Gene Ontology (BiNGO) plug-in in Cytoscape software. BiNGO provides *p-value *statistics based on the probability of occurrence of the genes/proteins in the defined ontological categories [31]. Here, the *p-values *for each disease biomarkers were calculated on the entire ontological categories such as molecular function, biological processes and cellular localization. Further, the geometric mean of *p-values *of each disease was calculated and the negative logarithm was performed. The biomarkers relationship to its disease was significant, if the score obtained to be greater than a threshold of 1.3 [32].

### Biomarker scoring for diagnosis from biofluid

The identified biomarkers were scored based on the feasibility of diagnosis. The biomarker score (Ψ_pi score_) for each protein (pi) was calculated by assigning the score for each parameter such as house keeping genes (μ_i_), urine protein (αi), plasma protein (βi) and CSF protein (γi) in a given scoring formula.

$$\Psi_{\text{pi score}}=\mu_{\text{i}}+\text{3}(\alpha \text{i}+\beta \text{i}+\gamma i)$$

The proteomic data of urine was obtained from [33] and plasma proteome data was obtained from the Human proteome organization database [34]. The CSF proteome and house keeping genes data were obtained from the literature of previous studies [35,36]. In scoring formula (Ψ_pi score)_, μ_i_: scored 1.0, if the protein (pi) is encoded by house keeping gene, else it is scored 0.5; αi = 0.3, if the protein (pi) circulating in CSF; βi = 0.5, if protein (pi) circulating in plasma; γi = 0.7, if the protein(pi) circulating in urine. The absence of protein (pi) in any biofluid indicated as, αi (or) βi (or) γi = 0.

### Cross validation of network

In order to validate our computational approach, the results obtained from this study were compared with the results of previous studies. The disease-disease interaction was cross-validated with Goni et al and Goh et al approaches [19,4]. Furthermore, the identified biomarkers were validated using Genome Wide Association studies (GWAS) database [23] to calculate the *precision rate*.

$$\text{Precision rate }(\text{PPV})\%=\frac{\text{TP}}{\text{TP}+\text{FP}}$$

TP: Number of True Positive

FP: Number of False Positive

GWAS contains disease associated gene/protein information in terms of gene expression, proteomic expression and mutation data. Cross validation of identified biomarkers with GWAS database will be valuable, to utilize the measurable threshold of our biomarkers for diagnosis.

## Authors' contributions

SSJ designed the study, performed the analyses, interprets the results and wrote the manuscript. ARA, SK and SC were contributed for data interpretation. WS is the principal investigator of this project. All authors have read and approved the final manuscript.

## Supplementary Material

Additional file 1 **MeSH based disease categorization**. Classification of manually collected 124 diseases based on the MeSH terms. This file is in *PSI-MI level 2.5 *format and can be viewed by Cytoscape software. Click here for file

Additional file 2 **Curated disease protein network (DPN)**. Disease protein network of 3632 proteins with 6172 interactions. This file is in *PSI-MI level 2.5 *format and can be viewed by Cytoscape software. Click here for file

Additional file 3 **Extracted disease-disease network (DDN) using scoring algorithm**. List of positively scored disease-disease interactions. This file is in *PSI-MI level 2.5 *format and can be viewed by Cytoscape software. Click here for file

Additional file 4 **Disease and aging interaction**. Positively scored interaction between of disease and aging. This file is in *PSI-MI level 2.5 *format and can be viewed by Cytoscape software. Click here for file

Additional file 5 **MeSH validated disease interaction pairs**. List of MeSH validated disease interactions. This file is in *PSI-MI level 2.5 *format and can be viewed by Cytoscape software. Click here for file

Additional file 6 **Common pathway network**. Common pathway associated disease pairs. This file is in *PSI-MI level 2.5 *format and can be viewed by Cytoscape software. Click here for file

Additional file 7 **Gene/protein sets uniquely representing specific disease as biomarkers**. Disease specific biomarker proteins. This file is in *PSI-MI level 2.5 *format and can be viewed by Cytoscape software. Click here for file
