# Supplementary material for: Systems biological approach on neurological disorders: a novel molecular connectivity to aging and psychiatric diseases
Source: BMC Syst Biol. 2011 Jan 12;5:6. doi: 10.1186/1752-0509-5-6 (PMC3033822; doi:10.1186/1752-0509-5-6)
Supplement: Additional file 1 — MeSH based disease categorization. Classification of manually collected 124 diseases based on the MeSH terms. This file is in PSI-MI level 2.5 format and can be viewed by Cytoscape software. [file 1752-0509-5-6-S1.RTF]

===============================================================

Psychiatric Disorders

===============================================================

Anxiety Disorders:
Agoraphobia

Panic Disorder

Impulse Control Disorders:
Pathologic Gambling

Trichotillomania

Mood Disorders:
Major Depressive Disorder

Manic Depressive Psychosis
Seasonal Affective Disorder

Schizophrenia and Disorders with Psychotic Features:
Capgras Syndrome

Schizophrenia
Graefe Disease

Sleep Disorders:
Cataplexy

Kleine-Levin Syndrome
REM Sleep Behavior Disorder
Restless Legs Syndrome
Sleep Bruxism
Sleep Deprivation
Sleep Paralysis
Sleep-Wake Transition Disorders
Somnambulism
Fatal Familial Insomnia

Somatoform Disorders:
Body Dysmorphic Disorder

Mental Disorders:
	Asperger syndrome
	Dyslexia
Obsessive-compulsive disorder


======================================================

Neurological Disorders

======================================================

Autoimmune Diseases of the Nervous System:
Encephalomyelitis

Lambert-Eaton Myasthenic Syndrome
Leukoencephalitis
Multiple Sclerosis

Autonomic Nervous System Diseases:
Adie Syndrome

Autonomic Dysreflexia
Causalgia
Dysautonomia
Multiple System Atrophy 

Central Nervous System Diseases:
Alzheimer Disease

Amblyopia
Amyotrophic Lateral Sclerosis
Angelman Syndrome
Ataxia Telangiectasia
Auditory Perceptual Disorder
Bardet-Biedl Syndrome
Basal Ganglia Hemorrhage
Cerebellar Neoplasms
Chorea
Chorea Gravidarum
Creutzfeldt-Jakob Syndrome
Dyslexia
Epilepsy
Essential Tremor
Fatigue Syndrome
Friedreich Ataxia
Frontotemporal Dementia

Gerstmann-Straussler-Scheinker Disease
Glycogen Storage Disease
Huntington Disease
Lafora Disease
Landau-Kleffner Syndrome
Lesch-Nyhan Syndrome
Leukoencephalitis
Multiple System Atrophy
Niemann-Pick Disease
Pantothenate Kinase-Associated Neurodegeneration
Paraparesis
Parkinsons Disease
Refsum Disease
Sandhoff Disease
Scrapie
Seizures
Stroke
Supranuclear Palsy
Tay-Sachs Disease
Torticollis
Tourette Syndrome
Vasospasm

Cranial Nerve Diseases:

Accessory Nerve Diseases 
Adie Syndrome

Facial Neuralgia 
Glossopharyngeal Nerve Diseases 
Vocal Cord Paralysis

Wolfram Syndrome 
Trochlear nerve disease

Demyelinating Diseases:
Alexander Disease

Canavan Disease
Multiple Sclerosis

Nervous System Malformations:
Acrocallosal Syndrome

Alstrom Syndrome
Charcot-Marie-Tooth Disease
Giant Axonal Neuropathy
Refsum Disease
Septo-Optic Dysplasia
Spastic Paraplegia
Tuberous Sclerosis

Nervous System Neoplasms:
Cerebellar Neoplasms

Neurocutaneous Syndromes:
Ataxia Telangiectasia

Tuberous Sclerosis
von Hippel-Lindau Disease

Neurodegenerative Diseases:
Alexander Disease

Alstrom Syndrome
Alzheimer Disease
Amyloid Neuropathy
Amyotrophic Lateral Sclerosis
Bulbar Palsy
Bulbo-Spinal Atrophy
Canavan Disease
Charcot-Marie-Tooth Disease
Friedreich Ataxia
Frontotemporal Dementia
Gerstmann-Straussler-Scheinker Disease
Giant Axonal Neuropathy
Huntington Disease
Lafora Disease
Lambert-Eaton Myasthenic Syndrome
Lesch-Nyhan Syndrome
Multiple System Atrophy
Pantothenate Kinase-Associated Neurodegeneration
Paraneoplastic Polyneuropathy
Parkinson Disease
Refsum Disease
Rett Syndrome
Scrapie
Spastic Paraplegia
Supranuclear Palsy
Tourette Syndrome
Tuberous Sclerosis
Wolfram Syndrome
ParaneoplasticCerebellar Degeneration

Neurologic Manifestations:
Ageusia
Amblyopia
Apraxia
Ataxia Telangiectasia
Athetosis

Auditory Perceptual Disorder
Brown-Sequard Syndrome
Catalepsy
Catatonia
Cerebrospinal Fluid Rhinorrhea
Chorea
Dyslexia
Facial Paralysis
Gait Apraxia
Gait Ataxia
Glycogen Storage Disease
Korsakoff Syndrome
Lesch-Nyhan Syndrome
Paraparesis
Paraplegia
Phantom Limb
Respiratory Paralysis
Rett Syndrome
Sciatica
Scotoma
Seizures
Supranuclear Palsy
Torticollis
Vertigo
Vocal Cord Paralysis
Wolfram Syndrome
Agnosia
Anisocoria
Cerebrospinal Fluid Otorrhea
Persistent Vegitative State

Neuromuscular Diseases:
Alstrom Syndrome

Amyotrophic Lateral Sclerosis
Brachial Plexus Neuropathies
Bulbar Palsy
Bulbo-Spinal Atrophy
Carpal Tunnel Syndrome
Causalgia
Cervical Rib Syndrome
Charcot-Marie-Tooth Disease
Eosinophilia-Myalgia Syndrome
Fatigue Syndrome
Femoral Neuropathy
Fibromyalgia
Giant Axonal Neuropathy
Isaacs Syndrome

Lambert-Eaton Myasthenic Syndrome
Paraneoplastic Polyneuropathy
Peroneal Neuropathies
Radial Neuropathy
Radiculopathy
Refsum Disease
Sciatica
Spastic Paraplegia
Paralysis Familial Periodic
Paranoid Disorder
